# In Vitro and In Vivo Correlation of Colon-Targeted Compression-Coated Tablets

**DOI:** 10.1155/2016/5742967

**Published:** 2016-02-17

**Authors:** Siddhartha Maity, Amit Kundu, Sanmoy Karmakar, Biswanath Sa

**Affiliations:** ^1^Division of Pharmaceutics, Department of Pharmaceutical Technology, Jadavpur University, Kolkata 700032, India; ^2^Division of Pharmacology, Department of Pharmaceutical Technology, Jadavpur University, Kolkata 700032, India

## Abstract

This study was performed to assess and correlate in vitro drug release with in vivo absorption of prednisolone (PDL) from a colon-targeted tablet prepared by compression coating of core tablet. In vivo drug absorption study was conducted using a high performance liquid chromatographic (HPLC) method, which was developed and validated for the estimation of PDL in rabbit plasma. The calibration curve showed linearity in the concentration range of 0.05 to 50 *μ*g/mL with the correlation coefficient (*r*) of 0.999. The method was specific and sensitive with the limit of detection (LOD) and lower limit of quantification (LLOQ) of 31.89 ± 1.10 ng/mL and 96.63 ± 3.32 ng/mL, respectively. The extraction recovery (ER) of PDL from three different levels of quality control (QC) samples ranged from 98.18% to 103.54%. In vitro drug release study revealed that less than 10% drug was released in 6.34 h and almost complete (98.64%) drug release was achieved in the following 6 h. In vivo drug absorption study demonstrated lower values of *C*_max_, AUC_total_, and protracted *T*_max_ from compression-coated tablet. The results confirmed the maximum release of drug in the colon while minimizing release in the upper gastrointestinal tract (GIT). An excellent in vitro and in vivo correlation (IVIVC) was also achieved after considering the lag time.

## 1. Introduction

In recent years, colon-targeted oral drug delivery systems have been investigated extensively to achieve better therapeutic response of anticancer, anti-inflammatory, steroidal, and anthelmintic drugs, which are used in various colon-related diseases [1]. The most important advantage of colon-targeted drug delivery system is to provide a high concentration of therapeutic agent at the site of action while minimizing premature drug release in the upper gastrointestinal tract (GIT), namely, stomach and small intestine, and thus reducing the emergence of adverse effects to nontarget areas [2]. Different technologies based on site-specific triggering have been developed to drive the drug molecules to the colon bypassing the upper GIT and to provide a sigmoidal drug release pattern involving a longer lag time (*T*_lag_) followed by burst release in the colon. The approaches include coating with pH-sensitive polymers, time dependent release systems, and compression coating with polysaccharides [3].

Several pH-sensitive microspheres dosage forms for colon targeting of drugs have been reported. pH-sensitive poly(3-hydroxybutyrate) based microspheres blended with cellulose acetate phthalate (CAP) [4] and polyethylene glycol cross-linked chitosan microspheres coated with CAP [5] have been reported to bypass the release of 5-fluorouracil (5-FU), an anticancer drug, in the gastric acidic environment and to provide slow release in intestinal condition. Polyhydroxybutyrate blended with CAP microsphere has also been found to provide prolongation of cytotoxic effect of 5-FU [6].

However, pH-sensitive and time dependent release systems exhibit unpredictable site specificity, respectively, because of large inter- and intrasubject variation and almost similar pH values of small intestinal and colonic fluids [7] and wide variation in gastric retention time [8]. Among the various technologies, compression-coated systems based on natural polysaccharides appear to be promising because they are degraded by the enzymes produced by the anaerobic microflora of colon [7, 9].

Natural polysaccharides have been used extensively in designing colon-targeted tablet dosage forms because they are biocompatible and biodegradable [10], highly stable, safe, nontoxic, and hydrophilic and fall under the category “generally regarded as safe” (GRAS) [11, 12]. Additionally, chemical modifications impart many important functionalities over the native polysaccharides for diverse application [13]. Frequently, a blend of polysaccharides provides more desirable drug release profile than a single polymer [14, 15]. Several polysaccharides such as guar gum, pectin, sodium alginate, and locust bean gum which remain undigested in the upper GIT but degrade by the enzymes secreted by colonic microflora have found applications in the formulation of compression-coated tablets [12, 16].

It has, however, been reported that the composition of human gut ecosystem may vary depending on the age, geographic provenance, dietary habit, disease, and intake of antibiotics and probiotics [17]. Moreover, degradation of certain polysaccharides like xanthan gum by colonic bacteria is questionable due to rigid structural framework [18–20]. Hence, it is rational to design a colon-targeted tablet by compression coating with polysaccharides that erodes slowly enough to retard premature drug release in the upper GIT and then provides burst release of the drug from the core tablet in the colon in the absence of colonic bacterial enzymes.

Xanthan gum, an exopolysaccharide obtained from* Xanthomonas campestris*, chemically consists of *β*,1-4-D-glucose backbone and a trisaccharide side chain consisting of two mannose residues separated by a glucuronic acid, attached with alternate glucose residues. The terminal D-mannose residue may contain a pyruvate group and the mannose closest to the backbone contains an acetyl function [21]. Sustained release of drugs from native xanthan gum tablets is well documented [21, 22]. We previously reported that matrix tablets composed of Ca^+2^ ion cross-linked carboxymethyl xanthan gum retarded the initial release of prednisolone for a considerable period of time, although complete drug release even in 10 h was not achievable [23]. Subsequently, we developed a compression-coated tablet in which core tablet of prednisolone containing microcrystalline cellulose (MCC, 55 mg), crospovidone (CP, 9 mg), trisodium citrate (TSC, 10 mg), and prednisolone (PDL, 15 mg) was coated with 225 mg of a blend of carboxymethyl xanthan gum (CMXG) and sodium alginate (SAL) in a ratio of 1.5 : 3.5, and the resulting tablet provided *T*_lag_, the time required to release 10% or less drug, of 6.06 h followed by a pulse release within 4.36 h, and, thus, the optimized tablet appeared suitable for colon specific delivery of PDL without the intervention of colonic bacterial enzymes in dissolution fluid [24].

Therefore, the objective of this study was to assess and correlate preclinical pharmacokinetic profiles of PDL with in vitro release from the compression-coated tablet. In order to facilitate oral administration of the tablet in rabbit, a minitablet having the same composition of the previously optimized tablet was prepared and was subjected to in vitro drug release study under dynamic pH shift condition and in vivo drug absorption study on rabbit's model. An HPLC method was developed and validated to estimate the concentration of PDL obtained from rabbit's plasma.

Prednisolone (PDL), a synthetic glucocorticoid, is most widely used in the treatment of human ailment [25], such as rheumatoid arthritis, inflammatory bowel diseases, psoriasis, and asthma [26–28]. It is used for controlling the symptoms/inducing remission in both ulcerative colitis (UC) and Crohn's disease (CD) [29]. In spite of desired pharmacological responses, it also induces a larger number of multifarious adverse effects when absorbed from the upper GIT [30, 31] and hence appears to be a suitable drug for targeting into the colon [32].

## 2. Materials and Methods

### 2.1. Materials

Prednisolone (PDL) and dexamethasone as internal standard (IS) were obtained from Mepro Pharmaceuticals, Mumbai, India. Carboxymethyl xanthan gum (CMXG) having a degree of substitution 0.8 was synthesized in our laboratory. Sodium alginate (SAL), CaCl_2_, 2H_2_O (CaCl_2_), microcrystalline cellulose, PH 102 (MCC), polyplasdone XL (crospovidone, CP), trisodium citrate (TSC), magnesium stearate (MS), and trisodium orthophosphate dodecahydrate (TSP) were purchased commercially. Methanol (HPLC-grade) was obtained from Rankem Pvt. Ltd. Dimethyl sulfoxide (DMSO), ethyl acetate (EA), ammonium acetate (AA), formic acid (FA), polyethylene glycol (PEG-400), and EDTA were purchased from Merck Specialties Pvt. Ltd., Mumbai, India. HPLC-grade Milli-Q water was used throughout the study. All other reagents and solvents of analytical grade were used as received.

### 2.2. Animals

The in vivo absorption study was conducted on 18 adult healthy male New Zealand rabbits weighing 1.5–2.0 Kg. The study was carried out as per the standard guidelines of “Committee for the Purpose of Control and Supervision of Experiments on Animals (CPCSEA),” Ministry of Social Justice and Empowerment, Government of India, and was approved by the Institutional Ethics Committee, Department of Pharmaceutical Technology, Jadavpur University, Kolkata (Approved Protocol number AEC/PHARM/1407/2014). Rabbits were acclimatized with 12 h light and dark cycles for 15 days and were given free access to standard food and water* ad libitum*. Rabbits were divided into three groups each consisting of six animals (*n* = 6) and were kept in fasted state 24 h prior to the experiment. Group I animals were given 0.1 mL of intravenous bolus of PDL (10 mg/mL PDL in 50% v/v of PEG-400 in sterile water for injection). Group II and Group III animals received, respectively, an immediate release core tablet and a compression-coated tablet both containing 5 mg of PDL.

### 2.3. Preparation of Core and Compression-Coated Tablets

The core and compression-coated tablets were prepared using 1/3rd of the ingredients used in the formulation of the optimized tablet having a larger size [24]. Initially, immediate release core tablets having a crushing strength of about 4 Kg, the composition and physical characteristics of which are shown in Table 1, were prepared by directly compressing a blend of drug and excipients with 3 mm punch in a 10-station rotary minipress tablet machine (RIMEK, Karnavati Engineering Ltd., Gujarat, India).

Granules, the composition of which is shown in Table 2, were prepared by wet massing a blend of CMXG and SAL with required amount of CaCl_2_ solution. The resulting damp mass was passed through #22 BS screen (width of aperture 0.710 mm) and dried at 60°C to a residual moisture content of 2–4%. The compression-coated tablets having a crushing strength of about 6 Kg were prepared in the following way: core tablet was placed centrally in 40% of the granules kept in a 5.5 mm die and remaining 60% granules were placed over the core tablet and compressed into tablet using a flat face 5.5 mm punch in a 10-station rotary minipress tablet machine (RIMEK, Karnavati Engineering Ltd., Gujarat, India). Fifty core tablets and compression-coated tablets were prepared in duplicate.

#### 2.3.1. Evaluation of Physical Characteristics of Tablets

The weight variation and friability of both the core and compression-coated tablets were evaluated following the methods as described in Indian Pharmacopoeia [33]. Drug contents of the core tablets were determined as per the method described elsewhere [23].

#### 2.3.2. In Vitro Drug Release Study

In vitro drug release study was performed as per the method described previously [23]. Six compression-coated tablets were placed in 700 mL 0.1 (M) HCl solution (37 ± 0.5°C) of pH 1.2 contained in 6 vessels of USP-II dissolution rate test apparatus (TDP-06P, Electro Lab, Mumbai, India) and rotated with paddles at 100 rpm. The pH of the solution was increased after 2 h to 7.4 by adding 200 mL 0.2 (M) trisodium orthophosphate dodecahydrate. After an additional 3 h period, the pH of the solution was changed to 6.8 by adding 5 mL 2 (M) HCl. Aliquots were removed at predetermined times and replenished immediately after each withdrawal with the same volume of fresh media maintained at 37°C. The aliquots following suitable dilution were analyzed at 248 nm using Microplate Spectrophotometer (Multiskan Go, Thermo Scientific, USA). The amount of PDL released from the tablets was calculated using calibration curves drawn in the respective dissolution medium.

### 2.4. Bioanalytical Method Development

#### 2.4.1. Instrumentation and Chromatographic Conditions

The HPLC system (Shimadzu, Kyoto, Japan) consisted of a LC-20AD solvent delivery unit, a SPD-M20A photodiode array detector, and Rheodyne injector with a 100 *μ*L loop. Detection and quantification were performed using LC solution. Chromatographic separation was performed isocratically at a flow rate of 1.0 mL/min using a Phenomenex C18 column (particle size 5 *μ*m; 250 mm × 4.6 mm i.d.; Phenomenex, Torrance, USA) at 25°C. The mobile phase consisted of methanol and buffer (5 mM ammonium acetate and 0.1% formic acid in Milli-Q water, pH 3.0) in a volume ratio of 58 : 42. 40 *μ*L of sample was injected into the loop of injector and the eluted peaks were measured at 245 nm using UV detector.

#### 2.4.2. Preparation of Stock and Working Solutions

Stock solutions of PDL and IS were prepared at a concentration of 2 mg/mL in DMSO and were stored at 2–8°C until being used. The working stocks of PDL were prepared from 2 mg/mL stock of PDL in DMSO by diluting the stock solution with 50% v/v DMSO solution in Milli-Q water afresh before use. The working stock (25.00 *μ*g/mL) of IS in ethyl acetate was also prepared from the stock solution of IS in DMSO (2 mg/mL).

#### 2.4.3. Preparation of Calibration Standards and Quality Control (QC) of Samples

In order to construct calibration curve, eleven calibration points in the analytical ranges from 0.05 to 50.00 *μ*g/mL of PDL with a fixed concentration of IS at 83.33 *μ*g/mL were selected. 10 *μ*L aliquot of PDL working solution (spiking solution) was spiked with 90 *μ*L of blank plasma and 500 *μ*L of IS (25 *μ*g/mL) in ethyl acetate (EA). The samples of spiked plasma were vortexed for 5 min for complete extraction of PDL and IS in EA fraction, centrifuged (RMI2C, Remi Cooling Centrifuge, Mumbai, India) at 7000 rpm for 10 min and allowed to stand for 30 min. The supernatant EA fraction was collected carefully and evaporated to dryness under a stream of nitrogen. The residues were reconstituted with 150 *μ*L of freshly prepared mobile phase. Finally, the samples were filtered through 0.2 *μ*m syringe filter, and 40 *μ*L was injected into the HPLC system. Three levels of QC samples at lower, middle, and higher concentration (LQC, MQC, and HQC), for example, 0.150 *μ*g/mL (LQC), 20.00 *μ*g/mL (MQC), and 40.00 *μ*g/mL (HQC), were also prepared in a similar way.

### 2.5. Bioanalytical Method Validation

The method was validated for specificity, linearity, accuracy and precision, extraction recovery, and stability according to the guidelines and protocols of the United States Food and Drug Administration [34].

#### 2.5.1. Specificity

The determination of specificity was performed by comparing the chromatograms of sample containing analyte (PDL) and IS against the blank plasma spiked with IS.

#### 2.5.2. Linearity

The linearity of calibration curve was assessed by eleven different concentrations of analyte (PDL) ranges from 0.05 to 50.00 *μ*g/mL with a constant concentration of IS (83.33 *μ*g/mL) in spiked plasma samples. Peak area ratios for each concentration level of analytes to IS were measured in six replicates (*n* = 6) and the calibration curve was constructed from the least square linear regression analysis. The linearity was represented as correlation coefficient (*r*).

#### 2.5.3. Accuracy and Precision

To determine the accuracy and precision, three QC samples (LQC, MQC, and HQC) were analysed for three consecutive days. Precision and accuracy were expressed in terms of coefficient of variation (% CV) and relative error (% RE), respectively. In case of precision, the values of CV ≤ 15% for MQC and HQC and CV ≤ 20% for LQC are acceptable. Similarly, in case of accuracy, the values of RE ≤ 15% for MQC and HQC and RE ≤ 20% for LQC are acceptable [34].

#### 2.5.4. Sensitivity

The limit of detection (LOD) and lower limit of quantification (LLOQ) were determined according to the following equation:

(1) $$\begin{array}{c} \text{LOD or LLOQ}=\delta \left(\;\frac{{\text{S}}_{\text{D}}}{S}\;\right), \end{array}$$

where *δ* is a constant (3.3 for LOD and 10 for LLOQ), S_D_ is the standard deviation of the analytical signal, and *S* is the slope of the concentration versus response graph.

#### 2.5.5. Extraction Recovery

The extraction recovery (ER) of analyte (PDL) at three different levels of QC samples (*n* = 6) was evaluated by comparing the peak area responses from the plasma samples spiked with analyte before extraction with those from blank plasma samples extracted and spiked with the same concentration of analyte after extraction. Similarly, the extraction recovery for IS was also performed for a particular concentration of 83.33 *μ*g/mL.

#### 2.5.6. Stability

Blank plasma, spiked with three different levels of QC samples, namely, LQC (0.150 *μ*g/mL), MQC (20.00 *μ*g/mL), and HQC (40.00 *μ*g/mL), was stored at different conditions: at room temperature for 24 h for short term, −20°C for one month and 3 months for long term, and 3 cycles for freeze-thaw stability studies. Area under the curves (AUCs) of the three levels of QC samples were measured.

### 2.6. In Vivo Absorption Study

Blood samples (0.5 mL) after intravenous (IV) bolus and oral administration of the respective formulations were collected carefully from the rabbit's marginal ear vein at 5, 15, 30, 60, 90, 120, 180, and 240 min intervals for Group I, at 5, 15, 30, 60, 120, 180, 240, 300, and 360 min intervals for Group II, and at 5, 15, 30, 60, 120, 240, 360, 420, 480, 540, 600, 630, 660, and 720 min intervals for Group III animals. The samples were collected in 1.5 mL microcentrifuge tube (Eppendorf, USA) containing 10% (w/v) of EDTA solution, immediately centrifuged at 3000 rpm for 5 min at 15°C in a Cold Centrifuge (Heraeus Megafuge 1.0R, Thermo Scientific, USA), and the supernatant plasma layers were separated and stored at −4°C until being used. The pharmacokinetic parameters including maximum plasma concentration (*C*_max_), the time required to reach *C*_max_ (*T*_max_), and mean residence time (MRT) were calculated using a software package (Kinetica 5.1).

### 2.7. In Vitro and In Vivo Correlation (IVIVC)

In vitro and in vivo correlation (IVIVC) is a predictive mathematical model, which describes the correlation between an in vitro (amount of drug released) and in vivo (amount of drug absorbed) results of a dosage form. Level A correlation is generally described as linear and represents a point-to-point relationship between in vitro drug release and the in vivo absorption of drugs. Level A IVIVC model using deconvolution method [35] has been adopted in this study design. In order to establish the IVIVC, percentage of drug absorbed in the systemic circulation after oral administration of various formulations was calculated based on the following model independent deconvoluted equation [36]:

(2) $$\begin{array}{c} C\left(\;t\;\right)=\;\overset{t}{\underset{0}{\int }}C_{\delta \text{iv}}\left(\;t-u\;\right)\cdot \Gamma_{\text{abs-vivo}}\left(\;u\;\right)\cdot du, \end{array}$$

where *C*(*t*) is the plasma concentration after oral administration of different tablets at time *t*, *C*_*δ*iv_ represents the plasma concentration after intravenous bolus injection, Γ_abs-vivo_ represents in vivo absorption rate, and *u* is the variable of integration.

## 3. Results and Discussion

### 3.1. Preparation, Evaluation, and Drug Release from Compression-Coated Tablet

In order to facilitate animal feeding, minicore (3 mm) and compression-coated (5.5 mm) tablets were prepared by 1/3rd reduction in the composition of the larger tablets optimized previously [24]. The immediate release core tablets and compression-coated tablets intended for colon specific delivery of PDL complied with the Pharmacopoeial requirement [34] with respect to weight variation, drug content, and friability (Tables 1 and 2).

**Figure 1:**
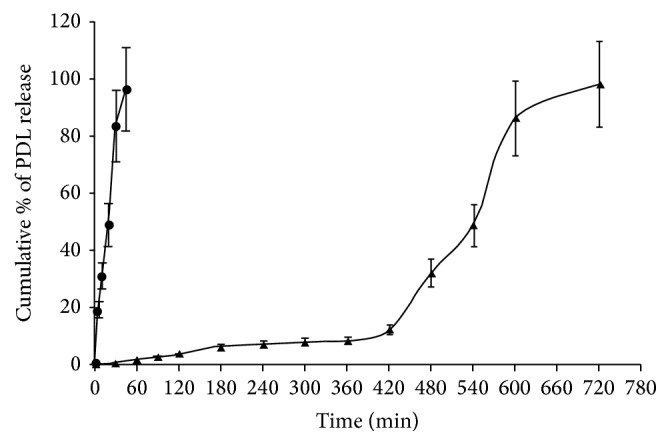
In vitro drug release profiles of immediate release core tablet (●) and compression-coated tablet (▲).

In vitro drug release studies were conducted in a condition mimicking the pH and transit time in GIT. Drug release from the core tablets was rapid and complete within 45 min (Figure 1) indicating that total amount of drug was released in gastric pH. On the other hand, only 3.9% and 8.69% drug were released from the compression-coated tablets, respectively, in 2 h and 6 h. In order to ascertain that drug release from the minitablet did not differ considerably from the previously optimized tablet having larger size, similarity (*f*_2_) and dissimilarity (*f*_1_) factors were determined and compared [37]. It was found that *f*_2_ value was 57.03 ± 1.02, whereas *f*_1_ value was 8.92 ± 0.41. This indicates that the release profile of tablet in reduced form did not change appreciably.

*T*_lag_, defined as the time required to release 10% or less drug, was found to be 6.34 h. During the next 6 h period almost complete (98.64%) drug release was achieved (Figure 1). *T*_rap_, the time required for rapid release following the lag time, was calculated by subtracting *T*_lag_ from the time required for complete release and was found to be about 6 h. This indicates an effective shielding of PDL release for an initial 6 h period during which the tablet may be located in the upper GIT and a rapid and complete release within the subsequent 6 h period when the tablet remains in the colon. Based on the results of in vitro drug release study it may be presumed that compression-coated tablets coated with a blend of Ca^+2^ ion cross-linked CMXG and SAL (1.5 : 3.5) might be suitable for colon targeting of PDL without the need of colonic bacterial enzymes.

### 3.2. Bioanalytical Method Development and Validation

**Figure 2:**
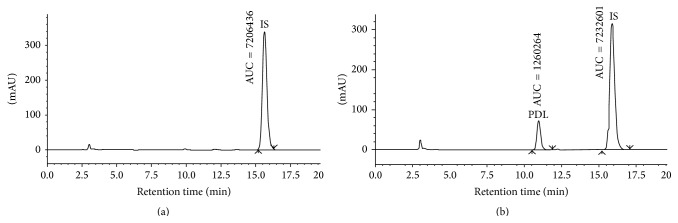
HPLC chromatogram of (a) plasma spiked with IS (83.33 *μ*g/mL) and (b) plasma spiked with PDL (25.00 *μ*g/mL) and IS (83.33 *μ*g/mL).

The HPLC method developed was sufficiently sensitive and suitable for estimation of PDL in rabbit's plasma. The specificity of an analytical method is the ability to differentiate and quantify the analyte (PDL) in the presence of any kind of interfering substances in the sample. The HPLC chromatograms of blank plasma spiked with IS and spiked with IS and PDL have been shown in Figure 2.

The retention time (*R*_*t*_) of IS and PDL varied from 15.86 ± 0.15 min to 15.98 ± 0.05 min and 10.91 ± 0.06 min to 11.00 ± 0.026 min, respectively. It was also noted that the chromatogram of PDL was not interfered by the endogenous substances of plasma as most of the interferences were found within 4 min. The calibration curve exhibited excellent linearity in the concentration range of 0.05 to 50 *μ*g/mL with correlation coefficient of 0.999. The calibration equation shows the average slope of 0.00693 (±0.00001, range: 0.00692 to 0.00694) and intercept of −0.000014 ± 0.000005.  Table 3 showed that all back calculated values of eleven calibration points were excellent in terms of accuracy (% RE) and precision (% CV).

The intraday and interday run precision (% CV) and accuracy (% RE) of PDL for three levels of QC sample (LQC, MQC, and HQC) ranged from 1.82 to 6.44% and −0.38 to 5.63%, respectively, and were within the acceptable limits (Table 4).

The limit of detection (LOD) and lower limit of quantification (LLOQ) were found to be, respectively, 31.89 ± 1.10 ng/mL and 96.63 ± 3.32 ng/mL indicating adequate sensitivity of the method for pharmacokinetic study. Moreover, the mean recoveries of PDL at LQC (0.150 *μ*g/mL), MQC (20.00 *μ*g/mL), and HQC (40.00 *μ*g/mL) samples were, respectively, 100.50 ± 1.34%, 98.22 ± 2.36%, and 103.77 ± 8.26%. The mean recovery of IS was 102.79 ± 3.79% of the concentration used in the assay procedure. Finally, the % CV and % RE under short term and long term stability studies varied from 1.67 to 7.30% and −0.56 to 4.14%, respectively, and were within the acceptable limits (Table 5).

### 3.3. In Vivo Drug Absorption Study

**Figure 3:**
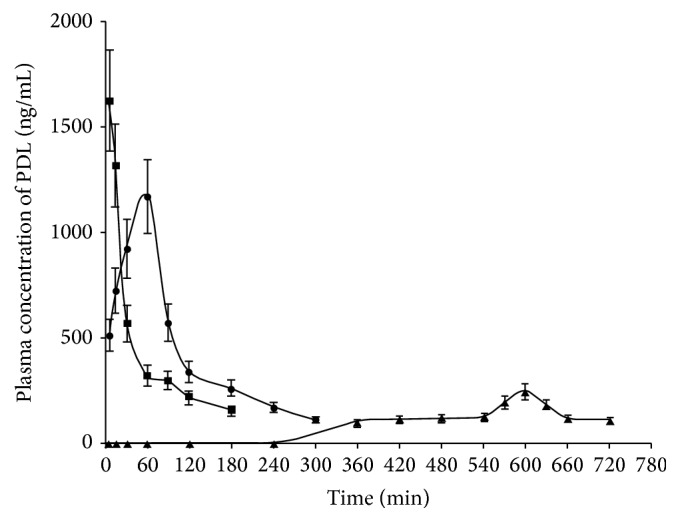
Plasma concentration versus time profiles of PDL obtained after oral administration of intravenous (IV) bolus administration (■), immediate release core tablet (●), and colon-targeted compression-coated tablet (▲).

Intravenous bolus injection of PDL was given in Group I animals to obtain data for in vitro and in vivo correlation (IVIVC). Immediate release core tablets and compression-coated tablets each containing 5 mg of PDL were given orally to Group II and Group III animals, respectively. The plasma concentration time profiles obtained following administration of the drug in different dosage forms are shown in Figure 3, and in vivo absorption parameters are depicted in Table 6.

The first sign of appearance of PDL in plasma in a concentration of 515.65 ± 4.48 ng/mL was recorded within 5 min following the administration of core tablets. The peak plasma concentration (*C*_max_) and the time (*T*_max_) required to reach *C*_max_ were, respectively, 1172.28 ± 22.98 ng/mL and 60 min. The drug concentration in plasma declined to 114.92 ± 6.28 ng/mL at the end of 5 h. On oral administration of the compression-coated tablets, quantifiable amount of PDL (100.42 ± 2.81 ng/mL) in plasma was found at 6 h. The plasma drug concentration increased slowly and *C*_max_ of 245.40 ± 10.42 ng/mL was reached at 10 h (*T*_max_) following which concentration declined and reached a level of 109.35 ± 4.29 ng/mL after 12 h. The results indicated that while PDL was rapidly absorbed from the stomach from the core tablets, compression-coated tablets released very small amount of drug in upper GIT within 6 h. This correlates well with the in vitro drug release where the drug tended to increase rapidly only after 6 h and almost complete drug release occurred within 12 h. Comparison of AUC_total_ values revealed that availability of the drug in systemic circulation from the compression-coated tablets was less than that from the core tablet. This was due to difference in anatomical region of drug release [38]. Compression-coated tablets released the drug in the colon as evident from considerable delay in the appearance of the drug in the plasma. Lower AUC_total_ value is an indication of reduced drug absorption from the limited absorptive surface of colon [39]. MRT of compression-coated tablets increased about 4 times in comparison to the core tablets suggesting that compression-coated tablet remained in the GIT for a prolonged period and did not expose the enclosed core tablet until it reached the colon.

### 3.4. In Vitro and In Vivo Correlation (IVIVC)

**Figure 4:**
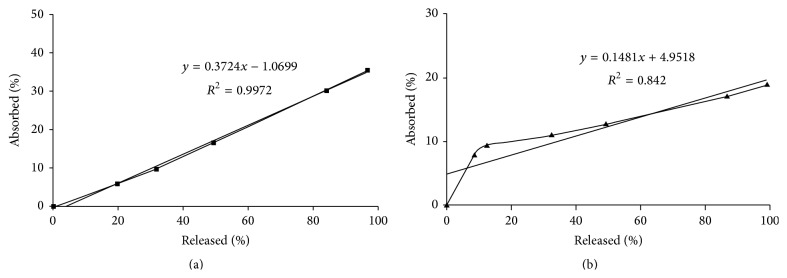
In vitro and in vivo correlation (IVIVC) of (a) immediate release core tablet and (b) colon-targeted compression-coated tablet.

To assess the correlation between in vitro drug release and in vivo drug absorption data, IVIVC study was carried out using immediate release core tablet and colon-targeted compression-coated tablet. When the cumulative percentage of drug released in vitro from immediate release core tablet was plotted against the percentage of drug absorbed in vivo, a good linear correlation (0.997) was observed (Figure 4).

In case of compression-coated tablet, when the cumulative percentage of drug released was plotted against the percentage of drug absorbed in vivo, a poor correlation (0.842) was observed. However, a good correlation (0.992) was observed when the cumulative percentage of drug released in vitro versus percentage of drug absorbed in vivo was plotted after considering the lag time of 360 min. Moreover, the IVIVC of colon-targeted compression-coated tablet appeared to be a hockey-stick curve that corresponds to nonlinear characteristics of drug release, and drug absorbed from the compression-coated tablet indicates that the mixed function of drug dissolution and permeation through colonic mucosa is the rate limiting step for drug absorption [40].

## 4. Conclusion

The results of this study indicated that in vitro release of PDL from the mini-compression-coated tablets was less than 10% in 6 h during which the tablets are supposed to be located in upper GIT and complete release was achieved in the following 6 h in the absence of colonic fluid. In vivo preclinical pharmacokinetic parameters determined by the validated HPLC method reflected the same pattern wherein the plasma concentration was considerably less in 6 h period and reached a maximum value in 10 h. The lower values of *C*_max_, AUC_total_, and protracted *T*_max_ in comparison to immediate release tablet indicated that PDL was released in the colonic region of rabbit with minimal drug release in the upper GIT from the compression-coated tablet. A good level A in vitro and in vivo correlation (IVIVC) was also achieved after the lag time of drug release in vitro and absorption in vivo. It may be concluded that a blend of natural and modified polysaccharides such as SAL and CMXG both cross-linked with Ca^+2^ ion to an optimum extent could be a suitable coating material for the development of colon-targeted tablets of PDL.

## Figures and Tables

**Table 1:** Composition and physical characteristics of core tablet.

Ingredients	Weight (mg)
PDL	5
MCC	18.33
CP	3
TSC	3.33
MS	0.34
Total	30

Physical characteristics	
Thickness (mm)	2.95 ± 0.06
Friability (%)	0.89%
Drug content (mg)	5.05 ± 0.25
Weight variation (%)	−4.08 to 4.84

**Table 2:** Composition and physical characteristics of compression-coated tablet.

Composition of coating material	Weight (mg)
CMXG	18.75
SAL	43.75
CaCl_2 _	12.50
Total	75

Physical characteristics of coated tablet	
Thickness (mm)	3.62 ± 0.04
Friability (%)	0.78
Weight variation (%)	−3.26 to 2.00

**Table 3:** Summary of the calibration standards at different levels of concentration.

Nominal concentration (*μ*g/mL)	Observed concentration (*μ*g/mL) (mean ± SD, *n* = 6)	% CV	% RE
50.00	50.296 ± 0.0077	0.02	0.59
25.00	25.124 ± 0.0037	0.01	0.50
12.50	12.559 ± 0.0031	0.02	0.48
6.25	6.271 ± 0.0021	0.03	0.34
3.125	3.145 ± 0.0007	0.02	0.64
1.56	1.568 ± 0.0011	0.07	0.54
0.80	0.785 ± 0.0002	0.02	−1.82
0.40	0.395 ± 0.0008	0.20	−1.26
0.20	0.198 ± 0.0000	0.01	−0.81
0.10	0.100 ± 0.0001	0.13	0.02
0.05	0.050 ± 0.0004	0.74	−0.77

**Table 4:** Summary of the intraday (*n* = 3) and interday (*n* = 9) precision (% CV) and accuracy (% RE) of the three levels of quality control (QC) samples.

Nominal concentration (*µ*g/mL)	Mean observed concentration (*µ*g/mL)	% CV	% RE
1st day (*n* = 3)			
0.150	0.151 ± 0.0028	1.85	−0.38
20.00	19.45 ± 0.48	2.48	2.73
40.00	38.10 ± 2.41	6.31	4.76
2nd day (*n* = 3)			
0.150	0.151 ± 0.0028	1.83	−0.47
20.00	19.53 ± 0.75	3.83	2.33
40.00	40.46 ± 1.46	3.60	−1.15
3rd day (*n* = 3)			
0.150	0.151 ± 0.0038	2.54	−0.68
20.00	18.87 ± 1.11	5.88	5.63
40.00	38.78 ± 2.50	6.44	3.05
Interday (*n* = 9)			
0.150	0.151 ± 0.0027	1.82	−0.51
20.00	19.29 ± 0.78	4.03	3.56
40.00	39.11 ± 2.15	5.51	2.22

**Table 5:** Summary of the short term and long term stability study data in three different levels of QC samples.

Nominal concentration (*µ*g/mL)	Mean observed concentration (*µ*g/mL)	% CV	% RE
3 freeze/thaw cycles (*n* = 6)			
0.150	0.144 ± 0.01	7.30	4.14
20.00	20.11 ± 0.81	4.02	−0.56
40.00	37.93 ± 2.03	5.36	5.17
Room temperature at 24 h (*n* = 6)			
0.150	0.148 ± 0.0073	4.91	1.11
20.00	19.56 ± 1.33	6.80	2.19
40.00	40.80 ± 2.47	6.06	−1.99
1 month at −20°C (*n* = 6)			
0.150	0.153 ± 0.0026	1.67	−2.05
20.00	19.23 ± 1.33	6.91	3.83
40.00	39.32 ± 1.73	4.41	1.69
3 months at −20°C (*n* = 6)			
0.150	0.145 ± 0.0046	3.13	3.12
20.00	19.74 ± 0.93	4.71	1.31
40.00	38.59 ± 1.45	3.75	3.53

**Table 6:** In vivo absorption parameters of PDL from various dosage form.

Parameters	Intravenous (IV)	Core tablet	Compression-coated tablet
*C*_max_ (ng/mL)	1624.29 ± 15.22	1172.28 ± 22.98	245.40 ± 10.42
*T*_max_ (min)	5	60	600
AUC_total_ (min·ng/mL)	104537 ± 1292.80	146075 ± 4133.50	83926.37 ± 1469.03
MRT (min)	130.05 ± 2.25	138.33 ± 5.74	572.33 ± 7.90
